# Patient-centered medical home care access among adults with chronic conditions: National Estimates from the medical expenditure panel survey

**DOI:** 10.1186/s12913-018-3554-3

**Published:** 2018-09-27

**Authors:** Ziyad S Almalki, Nedaa A Karami, Imtinan A Almsoudi, Roaa K Alhasoun, Alaa T Mahdi, Entesar A Alabsi, Saad M Alshahrani, Nourah D Alkhdhran, Tahani M Alotaib

**Affiliations:** 1 Department of Clinical Pharmacy, College of Pharmacy, Prince Sattam Bin Abdulaziz University, Al-Kharj, Riyadh, Saudi Arabia; 2 Department of Clinical Pharmacy, College of Pharmacy, Umm Al-Qura University, Makkah, Saudi Arabia; 3 College of Pharmacy, Princess Nourah bint Abdulrahman University, Riyadh, Saudi Arabia; 4 Department of Pharmaceutical Science, College of Pharmacy, Umm Al-Qura University, Makkah, Saudi Arabia; 5 Department of Clinical Pharmacy, College of Pharmacy, Jazan University, Jazan, Saudi Arabia; 6 Department of Pharmaceutics, College of Pharmacy, Prince Sattam Bin Abdulaziz University, Al-Kharj, Riyadh, Saudi Arabia; 7 College of Pharmacy, Prince Sattam Bin Abdulaziz University, Al-Kharj, Riyadh, Saudi Arabia

**Keywords:** PCMH, MEPS, Care access, Chronic conditions

## Abstract

**Background:**

The Patient-Centered Medical Home (PCMH) model is a coordinated-care model that has served as a means to improve several chronic disease outcomes and reduce management costs. However, access to PCMH has not been explored among adults suffering from chronic conditions in the United States. Therefore, the aim of this study was to describe the changes in receiving PCMH among adults suffering from chronic conditions that occurred from 2010 through 2015 and to identify predisposing, enabling, and need factors associated with receiving a PCMH.

**Methods:**

A cross-sectional analysis was conducted for adults with chronic conditions, using data from the 2010–2015 Medical Expenditure Panel Surveys (MEPS). Most common chronic conditions in the United States were identified by using the most recent data published by the Agency for Healthcare Research and Quality (AHRQ). The definition established by the AHRQ was used as the basis to determine whether respondents had access to PCMH. Multivariate logistic regression analyses were conducted to detect the association between the different variables and access to PCMH care.

**Results:**

A total of 20,403 patients with chronic conditions were identified, representing 213.7 million U.S. lives. Approximately 19.7% of the patients were categorized as the PCMH group at baseline who met all the PCMH criteria defined in this paper. Overall, the percentage of adults with chronic conditions who received a PCMH decreased from 22.3% in 2010 to 17.8% in 2015. The multivariate analyses revealed that several subgroups, including individuals aged 66 and older, separated, insured by public insurance or uninsured, from low-income families, residing in the South or the West, and with poor health, were less likely to have access to PCMH.

**Conclusion:**

Our findings showed strong insufficiencies in access to a PCMH between 2010 and 2015, potentially driven by many factors. Thus, more resources and efforts need to be devoted to reducing the barriers to PCMH care which may improve the overall health of Americans with chronic conditions.

## Background

In the United States (U.S.), chronic conditions are among major causes of disability, mortality, and high medical costs [1–4]. It has been estimated that nearly half (50.9%) of U.S. adults live with at least one chronic condition, while 26% have two or more chronic conditions [5]. These conditions are responsible for 46% of all deaths among the U.S. population annually. Furthermore, the associated costs of these conditions are enormous and compromise the health of the U.S. [6] It was estimated that 86% of U.S. health care expenditures are correlated with the treatment of chronic conditions [7].

With the growing number of chronic conditions [8], the associated costs made by these conditions will continue to threaten the entire federal budget. Over the last three decades, several improvements have been implemented into U.S. law, but they all focused heavily on insurance reforms. These steps will not be adequate unless they are coupled with fundamental health care improvement efforts targeting the primary care practice [9]. To achieve this goal, more attention has been paid to replace the poorly coordinated, acute-focused, episodic primary care practice with a care that is continuous, comprehensive, patient-centered, coordinated, and accessible, and that provides communication and shared decision-making [10].

A recent, successful approach to improve the chronic care management is the patient-centered medical home (PCMH). The PCMH model is an innovative primary care delivery system that has served to improve the quality of care and to reduce medical costs. PCMH rearranges how primary care service is designed and delivered to the patients, with the prime focus on patient needs and preferences [11, 12]. Over the past few years, with the growing numbers of adults with chronic conditions, many healthcare stakeholders in the U.S. have adopted the PCMH to prevent or inhibit the progression of specific chronic conditions [12].

Several studies have demonstrated the ability of PCMH application in improving the primary care quality, safety, and efficiency across the U.S. Some studies, for example, have suggested that receiving PCMH care is associated with a decreased number of hospitalizations and emergency room visits [13–18]. Others have also identified improvements in the quality of health care after implementing PCMH care [17, 19, 20].

Despite growing evidence in the literature that supports the effectiveness of the PCMH in improving health care outcomes and reducing costs, the extent of the PCMH’s adoption in treating Americans with chronic conditions remains unknown. Therefore, the objective of this study is to describe, at the national level, the changes in receiving PCMH among adults suffering from chronic conditions and to identify predisposing, enabling, and need factors associated with accessing PCMH care.

## Methods

### Data source

We conducted an observational cross-sectional analysis of the 2010–2015 Medical Expenditure Panel Survey (MEPS). MEPS has been conducted by the Agency for Healthcare Research and Quality (AHRQ) since 1996. MEPS is a nationally representative population-based survey of health care utilization and expenditures of the U.S. civilian noninstitutionalized population. The MEPS utilizes an overlapping panel design in which participant data are collected over a series of five rounds of interviews spaced about five months apart. The collected data include patient demographics, access to health care, use of health services, health conditions, health status, and other data as well. Information regarding the data and a description of its survey design have been published previously [21].

### Study population

Individuals aged 18 years and older who were diagnosed with at least one of the most common chronic conditions (i.e., hypertension, hyperlipidemia, mood disorders, diabetes, anxiety disorders, upper respiratory conditions, arthritis, asthma, or coronary artery disease) were identified. These conditions were considered to be chronic because they are long-lasting, cause diminished physical and/or mental capacity, or require long-term monitoring and medical interventions [22]. The prevalence of these conditions has been confirmed by the most recent data published by the Agency for Healthcare Research and Quality (AHRQ) [23]. According to MEPS documentation, patients in each year may be used as independent observations since each year in MEPS data is intended to be nationally representative [24].

### Primary outcome

The primary outcome of our analysis was determining whether the individual was receiving care consistent with PCMH principles. PCMH care was defined using the provider-related questionnaires in MEPS. AHRQ’s definition classifying PCMH care was used to determine whether respondents had a PCMH [25]. The respondent was considered to be receiving PCMH if the patient received comprehensive, patient-centered, and accessible care. Table 1 shows the survey items used to define PCMH features based on AHRQ’s criteria. Similar questions had been used in high-quality research to detect access to PCMH care using the same data [26–29].

**Table 1:** MEPS survey items used to define PCMH care

PCMH criteria	Survey items used
Comprehensive care	
	Does the provider usually ask about medications and treatments prescribed by other doctors
	Does the provider provide care for new health problems
	Does the provider provide preventive healthcare
	Does the provider provide referrals to other health professionals
	Does the provider provide care for ongoing health problems
Patient-centered care	
	Does the provider show respect for the medical, traditional, and alternative treatments other doctors may give
	Does the provider explain all healthcare options to participant
	Does the provider ask participant to help decide treatment choice
Accessible care	
	Is it difficult to contact the provider by phone about a health problem during regular office hours
	Does the provider offer night and weekend office hours
	Does the provider speak the participant’s language or provide translation services

We determined that the care received by an individual was comprehensive care if the provider did all of the following: 1) usually asked about any medications prescribed by other doctors; 2) provided care for new health problems; 3) provided preventive care; 4) offered referrals to other health professionals; and 5) provided care for ongoing health problems. We considered the individual to have received patient-centered care if the provider 1) showed respect for the medical, traditional, and alternative treatments other doctors may give; 2) explained all healthcare options to the individual; and 3) asked the individual to help decide on treatment. We considered care to be accessible if the provider 1) was easy to contact by phone about a health problem during regular office hours; 2) offered night and weekend office hours; and 3) spoke the participant’s language or provided translation services. Participants with responses of *don’t know*, *refused*, or *not ascertained* to any question were excluded from the final dataset.

### Independent variables

By using the Andersen Behavioral Model [30] in the current analysis, we examined the effects of person-specific predisposing, enabling, and need factors on having a PCMH. Predisposing factors investigated in this study included age, sex, race, marital status, and education years. Enabling factors consisted of health insurance, employment status, family income, and census region. (Appendix A contains a list of states composing each region with demographic data.) [31] Our assessments of health needs were based on self-rated health status variables (*good/excellent* or *poor/fair*).

### Data analysis

Descriptive statistics were used to characterize and evaluate changes in annual percentage for individuals who had PCMH over the six-year pooled dataset. The number of those individuals and their weighted percentage were calculated. Rao–Scott chi-square (a design-adjusted Pearson chi-square test) [32] analyses were performed to examine significant subgroup differences across strata for the two groups (having PCMH and having no PCMH). Adjusted multiple logistic regression analyses were then conducted to assess predictors associated with having a PCMH. In all analyses, we control for age, sex, race, marital status, education years, health insurance type, employment status, family income, chronic conditions, and calendar year. The c-statistic was calculated for each model to assess the model’s practical ability for correctly discriminating an individual outcome (PCMH/ No PCMH). A model demonstrates a good discrimination when the c-statistic is > 0.7 and outstanding when > 0.9.

To adjust for the complex multistage survey design and nonresponse, the estimates that are calculated from the data sample were multiplied by person-specific sampling weights provided within the original datasets of MEPS. All analyses were conducted with the use of SAS 9.4 software (SAS, Cary, NC).

## Results

A total of 20,403 patients with chronic conditions were identified, representing 213.7 million U.S. lives between 2010 and 2015. Approximately 19.7% of the patients were categorized as the PCMH group at baseline who met all the PCMH criteria defined in this study. The proportion of adults with chronic conditions who received a PCMH decreased from 22.3% in 2010 to 17.8% in 2015. However, in 2012 there was an increase in the number to 23.31% (Table 2).

**Table 2:** Annual changes in individuals with chronic conditions^a^

Year	*N*	*N*, weighted, in million	No PCMH, % (95% CI)	PCMH, % (95% CI)
2010	1458	15.6	77.69 (74.73–80.64)	22.31 (19.35–25.26)
2011	2935	31.8	81.21 (78.91–83.51)	18.78 (16.48–21.26)
2012	3725	37.3	76.68 (74.42–78.94)	23.31 (21.05–25.57)
2013	3313	33.7	81.31 (79.05–83.57)	18.68 (16.42–20.94)
2014	3112	33.7	80.13 (77.91–82.35)	19.86 (17.64–22.08)
2015	5860	61.3	82.17 (80.37–83.97)	17.82 (16.02–19.62)

Abbreviations: CI, confidence interval

^a^Sample size (*N*) is unweighted; Percentage weighted using weights provided with 2010–2015 MEPS

Table 3 presents the results of the study population’s descriptive characteristics. Individuals aged between 41 and 65 were most likely to report that they had at least one chronic condition (49.5%). The overall sample was predominantly female (57.1%), white (79.5%), married (57.8%), educated beyond high school (59.6%), insured by private insurance (70.1%), employed (58.1%), from a family with a high level of income (42.1%), from the southern U.S. geographical region (38.2%), and in excellent/good perceived health (79.7%). Hypertension, arthritis, and hyperlipidemia were the most prevalent chronic conditions among the study sample, 47.4%, 44.9%, and 37.8%, respectively.

**Table 3:** Baseline characteristics of individuals with chronic conditions, by PCMH access

Characteristic			Has a PCMH				*P*
	Total		No		Yes		
	*N*	Weighted %	*N*	Weighted %	*N*	Weighted %	
	(*N* = 20,403; Weighted *N* = 213,733,954)		(*N* = 16,443; Weighted *N* = 171,600,510)		(*N* = 3960; Weighted *N* = 42,133,444)		
Predisposing							
Age (Years)							0.001
19 to 40	5423	26.3	4299	25.9	1124	28.3	
41 to 65	10,227	49.5	8213	49.2	2014	50.5	
66 and older	4753	24.1	3931	24.8	822	21.2	
Sex							0.012
Female	12,196	57.1	9926	57.6	2270	55.2	
Male	8207	42.8	6517	42.4	1690	44.7	
Race							0.8
Non-white	6834	20.4	5485	20.5	1349	20.3	
White	13,569	79.5	10,958	79.5	2611	79.6	
Marital Status							<.0001
Married	10,810	57.8	8508	56.7	2302	62.1	
Never Married	4272	18.4	3465	18.5	807	18.3	
Separated	5321	23.6	4470	24.7	851	19.5	
Education Years							0.001
< 12 Years	3505	14.1	2980	14.7	525	11.8	
12 Years	4764	26.2	3833	26.3	931	25.5	
> 12 Years	8876	59.6	6956	58.9	1920	62.6	
Enabling							
Health Insurance							<.0001
Any Private	12,422	70.1	9708	68.5	2714	76.6	
Public Only	6301	23.7	5319	25.04	982	18.2	
Uninsured	1680	6.2	1416	6.4	264	5.2	
Employment Status							<.0001
Employed	11,006	58.1	8656	57.01	2350	62.7	
Not employed	9336	41.8	7734	42.9	1602	37.2	
Family Income Categorical							<.0001
High	6515	42.2	5001	40.8	1514	47.6	
Middle	5913	28.4	4747	28.3	1166	28.8	
Poor/ Low	7975	29.4	6695	30.8	1280	23.6	
Census Region							<.0001
Midwest	4073	21.8	3175	20.9	898	25.5	
Northeast	3355	17.8	2538	16.7	817	21.9	
South	7872	38.2	6583	39.8	1289	31.8	
West	5103	22.2	4147	22.5	956	20.8	
Healthcare Need							
Self-Reported Health							<.0001
Excellent/Good	15,144	79.7	1,1957	78.4	3187	85.03	
Fair/Poor	4872	20.3	4157	21.6	715	14.9	
Chronic Conditions							
Hypertension	10,207	47.4	8350	48.1	1857	44.4	0.001
Hyperlipidemia	7732	37.8	6359	38.6	1373	34.5	0.0001
Mood Disorders	3902	20.4	3259	21.3	643	17.05	<.0001
Diabetes Mellitus	4474	19.1	3673	19.4	801	17.9	0.06
Anxiety Disorders	3589	19.4	2976	19.9	613	17.1	0.002
Upper Respiratory Conditions	7405	38.8	5888	38.03	1517	42.1	0.0005
Arthritis	9250	44.9	7682	46.3	1568	39.3	<.0001
Asthma	2557	12.2	2071	12.3	486	11.8	0.4
Coronary Artery Disease	2197	10.8	1787	11.05	410	9.8	0.04

PCMH indicates Patient-Centered Medical Home

Compared to those who did not receive a PCMH, those who received PCMH were more likely to be younger, male individuals (44.7% vs. 42.4%), married individuals (62.1% vs. 56.7%), employed (62.7% vs. 57.01%), from families with higher income levels (47.6% vs. 40.8%), covered by private insurance (76.6% vs. 68.5%), and in excellent/good perceived health status (85.03% vs. 78.4%). They were also more likely to have achieved a higher level of education (had more than 12 years of education, 62.6% vs. 58.9%), and less likely to be from the southern U.S. geographical region (31.8% vs. 39.8%).

In Table 4, we found that the odds ratios (*OR*s) for individuals 66 years and older of having access to PCMH were 0.8 (confidence interval [CI]: 0.67–0.95). Compared with married individuals, those who were separated had significantly lower odds of having access to PCMH (*OR* = 0.78; CI: 0.67–0.91). Compared with individuals who completed fewer than 12 years of education, those who had more than 12 years of education had significantly higher odds of having a PCMH (*OR* = 1.25; CI:1.05–1.48).

**Table 4:** Adjusted odds ratios of having access to PCMH care among adults with chronic conditions, 2010–2015^a^

Independent Variable	Has a PCMH		*OR* ^b^	95% CI		*P*
	No	Yes				
Predisposing	*N*	*N*				
Age (Years)						
19 to 40	4299	1124	1.00			
41 to 65	8213	2014	0.93	0.82	1.06	0.3
66 and older	3931	822	0.80	0.67	0.95	0.01
Sex						
Female	9926	2270	1.00			
Male	6517	1690	1.08	0.99	1.18	0.05
Race						
Non-white	5485	1349	1.00			
White	10,958	2611	1.003	0.88	1.13	0.9
Marital Status						
Married	8508	2302	1.00			
Never Married	3465	807	0.87	0.75	1.01	0.06
Separated	4470	851	0.78	0.67	0.91	0.001
Education Years						
< 12 Years	2980	525	1.00			
12 Years	3833	931	1.17	0.99	1.37	0.05
> 12 Years	6956	1920	1.25	1.05	1.48	0.01
Enabling						
Health Insurance						
Any Private	9708	2714	1.00			
Public Only	5319	982	0.71	0.63	0.81	<.0001
Uninsured	1416	264	0.73	0.59	0.91	0.005
Employment Status						
Employed	8656	2350	1.00			
Not employed	7734	1602	0.83	0.74	0.93	0.001
Family Income Categorical						
High	5001	1514	1.00			
Middle	4747	1166	0.89	0.77	1.03	0.1
Poor/ Low	6695	1280	0.67	0.57	0.78	<.0001
Census Region						
Midwest	3175	898	1.00			
Northeast	2538	817	1.11	0.89	1.39	0.3
South	6583	1289	0.64	0.52	0.78	<.0001
West	4147	956	0.76	0.61	0.96	0.02
Healthcare Need						
Self-Reported Health						
Excellent/Good	1,1957	3187	1.00			
Fair/Poor	4157	715	0.65	0.56	0.76	<.0001
Chronic Conditions (Yes vs No)						
Hypertension	8350	1857	0.90	0.80	1.01	0.09
Hyperlipidemia	6359	1373	0.88	0.79	0.98	0.02
Mood Disorders	3259	643	0.79	0.69	0.90	0.0006
Diabetes Mellitus	3673	801	0.95	0.83	1.07	0.4
Anxiety Disorders	2976	613	0.81	0.707	0.93	0.002
Upper Respiratory Conditions	5888	1517	1.14	1.01	1.28	0.02
Arthritis	7682	1568	0.78	0.70	0.87	<.0001
Asthma	2071	486	0.93	0.80	1.06	0.3
Coronary Artery Disease	1787	410	0.96	0.82	1.11	0.5

Abbreviations: PCMH indicates Patient-Centered Medical Home; CI, confidence interval

^a^Sample size (*N*) is unweighted; Percentage weighted using weights provided with 2010–2015 MEPS

^b^Adjusted Odds Ratio

The result shows that the most important driver of having a PCMH was health insurance status. Compared with individuals covered by private insurance, those with public insurance were 71% as likely to have access to PCMH, while the uninsured were 73% as likely to have access to PCMH. There was also a significant difference in the employment status. Unemployed individuals were less likely to have access to PCMH compared to employed individuals (*OR* = 0.83; CI: 0.74–0.93).

Significant differences in the family income were observed in relation to having PCMH access. Individuals who were living in a poor or low-income family were about 33% less likely to have a PCMH compared to those living with a family with a high income (*OR* = 0.67; CI: 0.57–0.78). Individuals living in the South and West were the most likely to not have access to PCMH compared to individuals living in the Midwest (South: *OR* = 0.64; CI: 0.52–0.78; West: *OR* = 0.76; CI: 0.61–0.96). The analyses also showed that individuals who reported having fair or poor health were negatively associated with having a PCMH compared to those who reported excellent or good general health (*OR* = 0.65; CI: 0.57–0.76). In this population, individuals with the chronic conditions hyperlipidemia, mood disorders, anxiety disorders, and arthritis were significantly associated with limited access to PCMH. However, individuals diagnosed with upper respiratory conditions were positively associated with having access to a PCMH. The c-statistics associated with these adjusted logistic models ranged between 0.71 and 0.86.

## Discussion

As the first national study to present the extent of access to PCMH among adults with chronic conditions and to identify potential drivers for its trends, this study attempts to address this gap in the literature. In this research, we examined the prevalence of adult patients with chronic conditions who accessed PCMH care over the six-year period in the U.S.

This study found only a small percentage of patients with chronic conditions had access to PCMH care with a decreasing trend during the study period. This may raise concerns as this vulnerable population typically requires comprehensive and continuous care by primary care providers to manage their chronic physical problems, especially when the number and complexity of care needs increase as the number of chronic conditions a patient has increases [33]. In terms of medical services, the average numbers of ambulatory and emergency department visits, inpatient stays, and number of prescribed medications were much higher among individuals who suffered from two or more chronic conditions compared to those with no chronic condition [34].

To better understand the characteristics and drivers of that observed trend in this population, we analyzed many factors and found several factors were associated with access to PCMH. A change in one of these factors can cause a change in the PCMH trend. The older adults (66 and older) were less likely than comparable younger adults [19 to 40] to have access to PCMH care. This finding is consistent with what has been reported by prior studies that older patients were less likely to have PCMH access [35]. This can be explained by the dynamic health status of such individuals who often use more than one healthcare provider with no one provider responsible for all care. Older patients with chronic conditions are usually heterogeneous in terms of number and severity of chronic conditions, health status, and risk of adverse events [36]. Thus, policy leaders should promote access to PCMH care among older patients with chronic conditions because it may help coordinate their complex medical needs, which would improve quality and health outcomes. This was confirmed in a prospective before-and-after study among seniors receiving a PCMH. That study reported that seniors who experienced PCMH care made fewer and less costly emergency department visits and had fewer hospitalizations [37].

Our findings also revealed that marital status is an important factor associated with access to PCMH. Being separated had the effect of decreasing the likelihood of having access to PCMH versus being married. Similar to previously published studies, this study showed that the separated patients were less likely to receive PCMH care, although the number was not significant [38]. Our findings showed a positive association between a higher level of education and having access to PCMH care. A possible explanation of this finding is that better-educated individuals typically have a higher impact on changing their economic barriers to have full access to PCMH care [39].

All enabling factors were significantly associated with the probability of having PCMH access. Individuals with private insurance, employed, and living in a high-income family were found to report better access to PCMH. These findings are consistent with the literature in that access to PCMH is limited due to financial barriers [40]. Therefore, policy makers and health care providers should pay special attention to these barriers as they may negatively affect health-related outcomes, and the effect is substantial, especially among individuals with chronic diseases. Our findings suggest that expanding health insurance coverage is not an adequate approach to increase access to such care, but policy makers should also improve the provided public health insurance coverage for this population to have better access to PCMH care [41].

Clearly, census region is also important. Individuals who resided in the South or the West were less likely to have access to PCMH. This is not surprising because of the considerable difference in socioeconomic status of the majority of people who live in the South or the West compared to those in other regions. For example, a higher proportion of the population in the South and the West are racially Hispanic and Black [42]. There is evidence in many studies that these groups tend to not seek care for their chronic conditions [43–46]. Furthermore, compared to those in other regions, people in the South or the West are more likely to be uninsured, hence, less likely to have access to PCMH [47].

By looking closely at the chronic conditions, we identified a lack of uniform access to PCMH care across chronic conditions. We found that hyperlipidemia, mood disorders, anxiety disorders, and arthritis were significantly associated with limited access to PCMH, yet patients with upper respiratory conditions had better access to the care. A possible explanation is that upper respiratory conditions are minor and very common [48, 49]; thus, patients often seek the primary care provider’s help instead of the emergency department’s help, which results in a lower cost in managing their conditions.

Despite the uniqueness of the information provided by MEPS on individuals’ socioeconomics, access to care, and others in the U.S., there are limitations to the interpretation of the results of this study. First, as noted above, MEPS data provide information on the civilian, noninstitutionalized population, and hence exclude individuals living in institutions, such as individuals in nursing homes and long-term care hospitals who live with broad arrays of chronic conditions. Second, the definition of PCMH used in this study was based on patient responses, which might be subject to recall bias; thus, our estimates may underrepresent actual PCMH use. Despite the limitations, this study provides an important overview of the access to PCMH in a nationally representative general population sample of the U.S.

More effort is needed to facilitate access to PCMH among those with chronic conditions. In the PCMH care model, the primary care health professionals provide labor-intensive work behind the scenes, and it should be compensated accordingly because the total PCMH care fees ultimately demanded by physicians exceed the avoided expense for chronic conditions. This will increase access to PCMH, improve the quality of care, and reduce the overall cost associated with chronic conditions considerably [50, 51].

## Conclusion

Despite general agreement about the importance of PCMH, our findings showed strong deficiencies in access to PCMH between 2010 and 2015 to be potentially driven by many factors. These findings serve as a sign for more general problems with access to appropriate care. Moreover, reduced access to comprehensive and continuous services such as PCMH care may exacerbate chronic conditions, leading to more emergency department visits and hospitalizations that might have been preventable, as was reported in the literature. Thus, more resources and efforts need to be devoted to reduce barriers to PCMH care across the U.S., which may improve the overall health of Americans with chronic conditions.

## Appendix

**Table 5:** Demographic data by state

	2017 Population	Sex		Race					
		Male	Female	Hispanic			Not Hispanic		
				White	Black or African American	Asian	White	Black or African American	Asian
United States	325,719,178	160,408,119	165,311,059	53,403,379	3673,214	1,081,490	203,948,942	43,738,256	21,101,628
Northeast Region	56,470,581	27,530,306	28,940,275	6,670,850	1,413,848	130,784	37,714,017	6,915,133	4,206,459
Connecticut	3,588,184	1,751,800	1,836,384	494,988	79,472	7401	2,459,296	399,168	190,313
Maine	1,335,907	654,520	681,387	19,619	1833	672	1,267,954	27,024	22,099
Massachusetts	6,859,819	3,330,365	3,529,454	663,031	147,199	12,577	5,064,022	550,067	515,303
New Hampshire	1,342,795	665,009	677,786	43,686	5339	1011	1,235,192	24,697	43,679
Rhode Island	1,059,639	514,991	544,648	129,144	30,302	2578	787,314	75,632	43,896
Vermont	623,657	308,256	315,401	10,773	1080	315	590,084	11,433	14,181
New Jersey	9,005,644	4,396,574	4,609,070	1,583,995	232,080	26,086	5,074,996	1,231,086	952,219
New York	19,849,399	9,637,462	10,211,937	2,972,074	744,422	63,004	11,249,519	3,080,220	1,914,601
Pennsylvania	12,805,537	6,271,329	6,534,208	753,540	172,121	17,140	9,985,640	1,515,806	510,168
Midwest Region	68,179,351	33,659,324	34,520,027	4,907,673	328,391	75,567	52,871,947	7,828,966	2,621,209
Illinois	12,802,023	6,292,478	6,509,545	2,059,344	92,288	26,288	8,033,680	1,907,543	792,728
Indiana	6,666,818	3,287,095	3,379,723	424,866	31,395	6099	5,394,727	699,635	182,314
Michigan	9,962,311	4,903,752	5,058,559	448,997	45,859	7872	7,688,615	1,490,926	373,137
Ohio	11,658,609	5,713,100	5,945,509	380,535	56,605	7623	9,443,607	1,616,217	319,890
Wisconsin	5,795,483	2,882,738	2,912,745	360,587	25,733	5206	4,803,844	417,245	190,977
Iowa	3,145,711	1,564,733	1,580,978	174,674	8476	2622	2,745,459	143,876	94,566
Kansas	2,913,123	1,451,956	1,461,167	320,506	16,978	4476	2,278,889	204,687	105,079
Minnesota	5,576,606	2,776,846	2,799,760	266,704	20,460	6451	4,570,571	414,490	318,572
Missouri	6,113,532	3,002,236	3,111,296	232,440	19,122	4914	4,977,790	774,014	154,207
Nebraska	1920,076	958,131	961,945	189,923	8429	2832	1,549,724	109,839	58,318
North Dakota	755,393	387,299	368,094	23,519	1594	574	652,943	27,037	15,402
South Dakota	869,666	438,960	430,706	25,578	1452	610	732,098	23,457	16,019
South Region	123,658,624	60,616,528	63,042,096	20,466,319	1,205,243	240,734	72,437,426	24,796,491	5,027,316
Delaware	961,939	465,514	496,425	74,221	12,835	1245	617,848	223,603	44,712
District of Columbia	693,972	329,199	364,773	60,912	13,196	1737	267,319	325,427	35,717
Florida	20,984,400	10,256,819	10,727,581	4,998,757	346,858	46,802	11,635,713	3,457,022	716,287
Georgia	10,429,379	5,075,507	5,353,872	862,177	113,757	15,801	5,667,431	3,381,501	488,821
Maryland	6,052,177	2,934,154	3,118,023	514,832	81,314	13,744	3,199,793	1,884,099	454,595
North Carolina	10,273,419	5001,438	5,271,981	821,416	104,603	17,794	6,654,534	2,311,221	353,769
South Carolina	5,024,369	2,437,687	2,586,682	245,815	31,699	4933	3,277,257	1,397,097	103,733
Virginia	8,470,020	4,166,727	4,303,293	692,903	79,560	19,440	5,438,214	1,730,600	659,457
West Virginia	1,815,857	898,620	917,237	26,126	2588	609	1,703,491	80,375	19,632
Alabama	4,874,747	2,359,836	2,514,911	183,434	18,971	3211	3,264,132	1,329,710	86,055
Kentucky	4,454,189	2,194,318	2,259,871	145,839	13,639	2959	3,844,055	410,166	83,722
Mississippi	2,984,100	1,445,878	1,538,222	78,571	12,356	1799	1,721,204	1,136,985	39,692
Tennessee	6,715,984	3,275,966	3,440,018	324,771	28,280	6655	5,070,645	1,189,264	148,743
Arkansas	3,004,279	1,476,064	1,528,215	209,703	9559	2977	2,230,512	487,523	58,286
Louisiana	4,684,333	2,289,446	2,394,887	211,356	27,336	4703	2,807,713	1,545,237	101,469
Oklahoma	3,930,864	1,947,562	1,983,302	360,519	20,791	5623	2,782,296	349,881	111,591
Texas	28,304,596	14,061,793	14,242,803	10,654,967	287,901	90,702	12,255,269	3,556,780	1,521,035
West Region	77,410,622	38,601,961	38,808,661	21,358,537	725,732	634,405	40,925,552	4,197,666	9,246,644
Arizona	7,016,270	3,488,301	3,527,969	2,030,058	68,296	35,498	3,976,031	360,278	284,344
Colorado	5,607,154	2,822,333	2,784,821	1,107,360	43,409	19,603	3,944,067	273,910	230,929
Idaho	1,716,943	860,458	856,485	198,805	4338	3542	1,441,202	19,658	37,789
Montana	1,050,493	528,956	521,537	32,730	1333	995	930,784	10,369	13,960
Nevada	2,998,039	1,503,749	1,494,290	791,040	38,478	24,854	1,556,233	304,546	303,310
New Mexico	2,088,070	1,034,144	1,053,926	952,789	20,820	9381	811,077	48,485	41,353
Utah	3,101,833	1,561,688	1,540,145	399,778	13,416	7644	2,494,166	50,068	103,551
Wyoming	579,315	295,438	283,877	52,917	1579	795	496,212	9466	8176
Alaska	739,795	386,792	353,003	41,519	4643	2596	493,807	34,833	60,211
California	39,536,653	19,647,553	19,889,100	14,316,549	459,987	416,190	15,638,899	2,551,034	6,388,282
Hawaii	1,427,538	716,087	711,451	101,593	8043	68,273	516,294	42,935	747,347
Oregon	4,142,776	2,052,989	2,089,787	492,326	17,686	12,830	3,264,775	113,156	240,501
Washington	7405,743	3,703,473	3,702,270	841,073	43,704	32,204	5,362,005	378,928	786,891

## References

[1] Kochanek KD, Murphy SL, Xu JQ, Tejada-Vera B. Deaths: Final data for 2014. National vital statistics reports; vol 65 no 4. Hyattsville, MD: National Center for Health Statistics. 2016. (accessed Feb 16, 2018). 27378572

[2] United Health Foundation. America’s Health Rankings. A call to action for individuals and their communities. 2012 edition. https://assets.americashealthrankings.org/app/uploads/ahr16-complete-v2.pdf. (accessed Feb 16, 2018).

[3] Rudegeair P. Americans living longer, with unhealthy lifestyles: Report. Reuters. Dec 11, 2012. http://www.reuters.com/article/ 2012/ 12/11/us-usa-health-rankings-idUSBRE8BA1D220121211. (accessed Feb 16, 2018).

[4] Centers for Medicare and Medicaid Services. Chronic conditions among Medicare beneficiaries, chartbook, 2012 edition. Baltimore. 2012. https://www.cms.gov/Research-Statistics-Data-and-Systems/Statistics-Trends-and-Reports/Chronic-Conditions/Downloads/2012Chartbook.pdf (accessed Feb 16, 2018).

[5] Ward BW, Schiller JS (2013). Prevalence of multiple chronic conditions among US adults: estimates from the National Health Interview Survey, 2010. Prev Chronic Dis.

[6] Centers for Disease Control and Prevention. Leading causes of death and numbers of deaths, by sex, race, and Hispanic origin: United States, 1980 and 2014 (Table 19). Health, United States, 2015. https://www.cdc.gov/nchs/data/hus/hus15.pdf. (accessed Feb 16, 2018).

[7] Facts F. National Center for Chronic Disease Prevention and Health Promotion. CDC. https://www.cdc.gov/chronicdisease/about/costs/. (accessed Feb 16, 2018).

[8] Freid VM, Bernstein AB, Bush MA (2012). Multiple chronic conditions among adults aged 45 and over: trends over the past 10 years. NCHS data brief, no. 100.

[9] Chin MH (2008). Improving care and outcomes of uninsured persons with chronic disease. Now. Ann Intern Med.

[10] Rittenhouse DR, Shortell SM, Fisher ES (2009). Primary care and accountable care – two essential elements of delivery-system reform. N Engl J Med.

[11] Braddock Clarence H., Snyder Lois, Neubauer Richard L., Fischer Gary S. (2012). The Patient-Centered Medical Home: An Ethical Analysis of Principles and Practice. Journal of General Internal Medicine.

[12] Rich EC (2012). Organizing Care for Complex Patients in the patient-centered medical home. Ann Fam Med.

[13] Palfrey, Judith S., et al. The pediatric Alliance for coordinated care: evaluation of a medical home model. Pediatrics, (2004);113:Supplement 4 1507-1516. 15121919

[14] Mainous AG (2005). Impact of providing a medical home to the uninsured: evaluation of a statewide program. J Health Care Poor Underserved.

[15] Cleave V (2016). Jeanne, et al. medical homes for children with special health care needs: primary care or subspecialty service?. Acad Pediatr.

[16] Cooley WC (2009). Improved outcomes associated with medical home implementation in pediatric primary care. Pediatrics.

[17] Rankin KM (2009). Illinois medical home project: pilot intervention and evaluation. Am J Med Qual.

[18] Roby DH (2010). Impact of patient-centered medical home assignment on emergency room visits among uninsured patients in a county health system. Med Care Res Rev.

[19] Reid RJ, Fishman PA, Yu O, Ross TR, Tufano JT, Michael P (2009). Patient-Centered Medical Home Demonstration: A prospective, quasi-experimental, before and after evaluation. Am J Manag Care.

[20] Jaén CR (2010). Patient outcomes at 26 months in the patient-centered medical home National Demonstration Project. Ann Fam Med.

[21] Ezzati-Rice T, Rohde F, Greenblatt J. Sample design of the medical expenditure panel survey household component, 1998–2007. Methodol Rep. 2008;22.

[22] Basu J, Avila R, Ricciardi R (2016). Hospital readmission rates in U.S. states: are readmissions higher where more patients with multiple chronic conditions cluster?. Health Serv Res.

[23] Gerteis, J., D. Izrael, D. Deitz, L. LeRoy, R. Ricciardi, T. Miller, and J. Basu, Multiple chronic conditions Chartbook, AHRQ publications no. Q14–0038, Rockville, Md.: Agency for Healthcare Research and Quality, 2014.

[24] Medical Expenditure Panel Survey. MEPS HC-036: 1996-2008 Pooled estimation file. Agency for Healthcare Research and Quality, Rockville, MD. Available at: http://www.meps.ahrq.gov/mepsweb/data_stats/download_data/pufs/h36/h36u08doc.shtml#30Survey. (accessed Feb 16, 2018).

[25] Agency for Healthcare Research and Quality. Defining the PCMH. http:// pcmh.ahrq.gov/page/defining-pcmh. (accessed Feb 16, 2018).

[26] Jones Audrey L., Cochran Susan D., Leibowitz Arleen, Wells Kenneth B., Kominski Gerald, Mays Vickie M. (2015). Usual Primary Care Provider Characteristics of a Patient-Centered Medical Home and Mental Health Service Use. Journal of General Internal Medicine.

[27] Beal A, Hernandez S, Doty M (2009). Latino access to the patient-centered medical home. J Gen Intern Med.

[28] Jerant A, Fenton JJ, Franks P (2012). Primary care attributes and mortality: a national person-level study. Ann Fam Med.

[29] Stockbridge EL, Philpot LM, Pagán JA (2014). Patient-centered medical home features and expenditures by Medicare beneficiaries. Am J Manag Care.

[30] Andersen RM (1995). Revisiting the behavioral model and access to medical care: does it matter?. J Health Soc Behav.

[31] US Census Bureau: Census Interactive Population Search, 2010. Available at: https://factfinder.census.gov/faces/tableservices/jsf/pages/productview.xhtml?pid=PEP_2017_PEPANNRES&src=pt. (accessed Sept 6, 2018).

[32] Sorace J (2011). The complexity of disease combinations in the Medicare population. Population Health Management.

[33] Rao JNK, Scott AJ (1987). On simple adjustments to Chi-Square tests with sample survey data. Ann Stat.

[34] Machlin SR, Soni A (2013). Health care expenditures for adults with multiple treated chronic conditions: estimates from the medical expenditure panel survey, 2009. Preventive Chronic Diseases.

[35] Tarraf W, Jensen G, González HM (2017). Patient centered medical home care among near-old and older race/ethnic minorities in the US: findings from the medical expenditures panel survey. J Immigr Minor Health.

[36] American Geriatrics Society Expert Panel on the Care of Older Adults with Multimorbidity (2012). Patient-centered Care for Older Adults with multiple chronic conditions: a stepwise approach from the American Geriatrics Society. J Am Geriatr Soc.

[37] Fishman PA (2012). Impact on seniors of the patient-centered medical home: evidence from a pilot study. The Gerontologist.

[38] Jones AL (2015). Usual primary care provider characteristics of a patient-centered medical home and mental health service use. J Gen Intern Med.

[39] Making Americans Healthier (2009). Social and economic policy as health policy. Health Aff.

[40] Reibling N (2016). The patient-centered medical home: how is it related to quality and equity among the general adult population?. Med Care Res Rev.

[41] DeVoe JE (2007). Insurance + access != health care: typology of barriers to health care access for low-income families. Ann Fam Med.

[42] Kaiser Family Foundation estimates based on the Census Bureau's March Current Population Survey (CPS: Annual Social and Economic Supplements), 2017.

[43] Kim G, Ford KL, Chiriboga DA, Sorkin DH (2012). Racial and ethnic disparities in healthcare use, delayed care, and management of diabetes mellitus in older adults in California. J Am Geriatr Soc.

[44] Brinjikji W, Rabinstein AA, Lanzino G, Cloft HJ (2012). Racial and ethnic disparities in the treatment of unruptured intracranial aneurysms: a study of the Nationwide inpatient sample 2001–2009. Stroke.

[45] Eapen ZJ, Al-Khatib S, Lopes RD, et al. Are racial/ ethnic gaps in the use of cardiac resynchronization therapy narrowing? An analysis of 107,096 patients from the national cardiovascular data registry’s ICD registry*.* J Am Coll Cardiol 2012;60:1577–1578. 10.1016/j.jacc.2012.06.024 22958954

[46] Joshi Shivam, Gaynor Jeffrey J., Bayers Stephanie, Guerra Giselle, Eldefrawy Ahmed, Chediak Zoila, Companioni Lazara, Sageshima Junichiro, Chen Linda, Kupin Warren, Roth David, Mattiazzi Adela, Burke George W., Ciancio Gaetano (2013). Disparities Among Blacks, Hispanics, and Whites in Time From Starting Dialysis to Kidney Transplant Waitlisting. Transplantation Journal.

[47] Kaiser Family Foundation analysis of the 2016 National Health Interview Survey. (accessed 17 Feb 2018).

[48] Rietveld RP, Bindels PJE, ter Riet G (2006). Antibiotics for Upper Respiratory Tract Infections and Conjunctivitis in Primary Care: Reconsideration of prescription policy is needed. BMJ: British Medical Journal.

[49] Spillman, Nancy Z. Science of Breath (1999). A Practical Guide.

[50] Hussey PS, Eibner C, Ridgely MS (2009). Controlling US health care spending—separating promising from unpromising approaches. N Engl J Med.

[51] Fields D, Leshen E, Patel K (2010). Analysis & commentary. Driving quality gains and cost savings through adoption of medical homes. Health Aff (Millwood).

